# The Hospital

**Published:** 1917-12-08

**Authors:** 


THE HOSPITAL, December 8, 1917.]

### THE INSTITUTIONAL WORKER

**Being a Special Supplement to "The Hospital"**

### OUR BUREAU OF INFORMATION.

### Rules for Correspondents.

1. Every letter must be accompanied by the coupon to be cut from the back cover (inside page) of THE HOSPITAL, current issue, and must contain the name and address of the correspondent with pseudonym for publication if desired. Replies by post cannot be given save under exceptional circumstances at the Editor's discretion.

2. Letters from Approved Homes in reply to special needs published in the Bureau should state terms and full particulars, and be sent prepaid under cover to the Editor of the Bureau with name written across coupon for identification.

3. Proprietors of Homes which have not yet been entered on the List of Approved Homes, but have spare accommodation likely to suit special needs, are invited to write for an application form for registration The fee for registration, which includes two announcements of the Home in the Bureau and other privileges is 10s.

4. All communications to be addressed to the Editor of THE HOSPITAL, 28 Southampton Street, Strand, London, W.C. 2, and marked "Bureau of Information."

### Starting an Auxiliary Military Hospital.

[In response to special enquiries we have pleasure in giving full lists of many requirements.—Ed.]

### The Staff and Equipment. (Continued from December 1,)

#### The Theatre.

As a rule only minor operations are undertaken in an auxiliary hospital, the more important cases being transferred to a military hospital.

A class-room with a good light, and electric light installed, makes a suitable theatre.

If possible, a terrazzo floor should be chosen, or a boarded floor covered with good linoleum would answer the purpose well.

The fitting-up of the theatre with appliances and instruments, etc., usually depends on the surgeon-in-charge, but as a general rule the following would be required :

##### FURNITURE.

- 1 operating table on stand with fittings.

- 2 operating stools (revolving).

- 1 instrument case on stand.

- 1 anæsthetist's table, with two glass shelves.

- 1 table with zinc top.

- 1 gas-ring.

- 1 copper kettle.

- 2 sterilisers for instruments, with wire tray.

- 1 towel and mackintosh rail.

- 1 hand lotion-bowl on stand.

- 2 porcelain lavatory basins, fixtures, with taps.

- 1 porcelain sink.

- 1 cupboard for drugs, etc.

##### APPLIANCES.

- 6 or 4 dressing drums.

- 4 large enamel jugs.

- 6 enamel bowls (large).

- 18 enamel bowls (small).

- 3 porcelain trays of different sizes for instruments.

- 2 kidney trays.

- 3 glass lotion-jars.

- 1 glass tubing-jar.

- 3 small glass jars for catgut.

- 1 irrigator.

- 1 bell lamp.

- 1 refuse tray.

- 2 dressing pails.

- 4 nailbrushes.

##### INSTRUMENTS.

- 24 pairs of artery forceps.

- 12 scalpels.

- 2 razors.

- 3 pairs scissors, two straight and one curved.

- 1 suture scissors.

- 2 pairs dressing forceps.

- 2 pairs sinus forceps.

- 1 pair instrument forceps.

- 6 pairs of towel clips.

- 2 probes.

- 2 probe directors.

- 4 pair tissue forceps.

- 6 retractors.

- 1 saw.

- 1 pair bone-cutting forceps.

- 1 pair sequestrum forceps.

- 1 gouge.

- 3 spoons or curettes of different sizes.

- 1 aneurism needle.

- 2 pairs swab holders.

- 1 chisel.

- 2 raspatories.

- 2 dissectors.

- 2 mask frames.

- 1 pair tongue forceps.

- 1 tongue depressor.

- 2 mouth gags.

- 1 stethoscope.

- 1 hypodermic syringe and six needles.

- 2 record syringes (10 c.c.).

- 1 saline infusion apparatus.

- 3 tourniquets.

##### DRUGS, ETC.

- 3 bottles anæs. ether.

- 1 bottle A.C.E. mixture.

- 2 bottles chloroform.

- 1 set of drop bottles.

- 1 bottle liq. morphia.

- 1 bottle liq. strychnine.

- 1 bottle liq. ether.

- 1 bottle liq. strych. c. digitalone.

- 1 bottle liq. pituitrin.

- 2 tubes novocaine.

- 1 bottle collodion.

- 1 bottle sol. of ammonia.

- 1 bottle pure carbolic acid.

- 1 bottle formalin (40 per cent.)

- 1 bottle Whitehead's varnish.

- 1 bottle atropin.

- 1 box amyl. nitrate capsules.

- 1 bottle iodine in spirit (3 per cent.).

- 1 bottle sol. for sterilising catgut.

- 1 bottle ethereal soap.

- 2 sprays ethyl chloride.

- 1 jar vaseline.

- 4 bottles biniodide lotion, 1-500.

- 2 bottles corrosive lotion, 1-500.

- 2 bottles boric lotion, 1-40.

- 2 bottles carbolic lotion, 1-20.

- 1 bottle turpentine.

- 1 jar glycerine.

- 1 bottle methylated spirit.

- 1 bottle izal.

- 2 oxygen cylinders.

##### SUNDRIES.

- 4 reels thread.

- 5 tins catgut, 00, 1, 2, 3, 4.

- 4 boxes silkworm gut (fine, medium, strong, extra strong).

- 3 glass measures (1 minim, 1 oz., 1 pint).

- Needles (straight, full curved, half curved, round eyed, spring eyed).

- Glass rods, tubing, nozzles.

- Rubber tubing, different sizes.

- Reels for thread and silk.

- Skeins of silk.

- 2 funnels.

- Surgical strapping, 1 in. by 3 in.

- 24 pairs of rubber gloves of different sizes.

- 6 gum elastic catheters.

- 6 Jacques' catheters.

- 1 rectal saline apparatus.

- 1 Higginson's syringe.

- Reels ribbon gauze.

- Gauze swabs (large number).

- Green protective tissue.

##### LINEN, ETC.

- 12 surgeon's overalls.

- 36 operation towels.

- 12 nurse's overalls.

- 6 towels.

- 3 mackintosh aprons.

- 12 surgeon's masks.

- 12 mackintoshes (1 yd. sq.).

##### STERILISING.

To save the expense of fixing up a sterilising apparatus in each auxiliary hospital, a steriliser is fixed at the principal military hospital for the district, and all the sterilising for the smaller hospitals is done there, an orderly taking the drums there each morning and fetching them at night. Where this arrangement is not possible a steriliser has to be fixed, which means an additional £100 expense to the equipment of the hospital.

*(To be continued.)*

### Question for December.

### A System of Time-keeping for the Nursing and Domestic Staff.

What is the most satisfactory method of checking the going out and coming in of the nursing and domestic staffs of an institution which has three outlets, none of which it is practicable to keep locked during the day, nor is it possible to keep porters on duty for the purpose?

RULES.

The following rules must be observed:—
1. Contributions must be written on one side of the paper. Brevity and terseness are desirable features. The MSS. must bear the name and address of the sender and be accompanied by coupon to be cut from the back cover (inside page) of the current issue of The Hospital. A pseudonym must be chosen if the name is not to be published.
2. Contributions must be addressed to the Editor of The Hospital, Institutional Worker Supplement, 28 & 29 Southampton Street, Strand, London, W.C. 2, should reach him before the end of the current month, and be marked in the left-hand corner "Facts and Figures."

**A minimum payment of Half-a-Guinea will be made for each published answer.**

### QUESTIONS INVITED.

In connection with our Question Box, we offer every month five shillings each for the two best questions which are sent in for consideration. The questions may be on any institutional subject and concern any department, from the secretary's to the porter's, or the matron's to the domestic staff; the one essential is that they must be practical and deal with points that have a definite relation to hospital or institutional administration, upkeep, finance, or management. Points relating to artificers' work, housekeeping, the laundry, out-patients, and other practical matters will be welcome. It is hoped that thus, when difficult or doubtful points arise in the course of their work, institutional workers will be encouraged to put them in the form of a question and send them to the Editor, The Hospital Bureau of Information, 28 Southampton Street, Strand, W.C. 2, marked "Question Box."

The best questions will be published in due course and our readers will be given the opportunity of answering them. Thus all institutional workers may be able to co-operate with the view of helping the work and smoothing the difficulties of each other. In short, we want the Question Box of the *Institutional Worker* to be freely used by every worker habitually.

### ENQUIRIES AND ANSWERS.

### *THE SICK AND IN NEED.*

### How to Obtain Artificial Limb for Poor Woman.

We suggest that you write to the Secretary, Surgical Aid Society, Salisbury Square, Fleet Street, E.C., on behalf of the poor woman who requires an artificial limb. Help is given free with subscribers' letters. Where the cost of any surgical appliance exceeds the value of one letter, a number of letters (varying from one up thirty) proportionate to the outlay will be required.—H. O.

### Medical Attendance for Poor Married Woman.

The Royal Maternity Charity of London, 31 Finsbury Square, E.C., provides midwives and medical attendance free to poor married women in their own homes. Insured cases are considered on their merits. Write to the Secretary, giving full particulars of the case.—Distressed.

### Help for Blind Woman.

The case is one which would be eligible for help by the Royal School for the Indigent Blind, Leatherhead, Surrey. This institution ministers to the wants of blind men and women under training in food, clothing, etc. Application should be made to the Secretary, Rev. St. Clair Hill.—Needy One.

### *THE HOUSEKEEPERS' DEPARTMENT.*

### Milk Jelly for Invalids.

For this you will require one plain jelly square (Chivers' are very good) and nearly one pint of new milk, which should be slightly warmed. Cut up the jelly square and put the pieces into a basin. Dissolve these by standing the basin in very hot water, and when almost cold pour the warm milk in very slowly, stirring all the time. Then turn into a wet mould. The jelly must only be slightly warm, or the milk will curdle.—Private Nurse.

### What to Use in Place of Currants and Sultanas.

You will find that crystallised fruits cut up into small pieces are an excellent substitute for currants, sultanas, etc. You do not require many fruits, so that the expense is really not great, especially as you only want to make a few extra cakes for the nurses' Christmas fare.—Housekeeper.

### Cooking of Green Vegetables.

In cooking spinach or any other green vegetable it is a good plan first to place the vegetable in plenty of very hot water, then throw it into cold water, afterwards boiling it in a little stock that you may have at hand. This method ensures the greens keeping an excellent colour.—M. O. M.

### *EMPLOYMENT AND TRAINING.*

### Cookery Certificate.

You should apply either to the National Training School of Cookery, Buckingham Palace Road, London, S.W., or the Victoria School of Cookery, 4 Onslow Place, South Kensington, London, S.W., with a view to obtaining your cookery certificate.—René.

### *MISCELLANEOUS.*

### Regulation of Temperature.

By the exercise of a little care there is no reason why you should not overcome the trouble you refer to of excessively hot or unduly cold wards. This is a matter we have referred to more than once in the columns of the "Bureau." You should instruct your stoker to take daily readings of the temperature outside the building, and to raise the heat of the circulating water in the radiators accordingly. As a rule a building can be maintained at a mean temperature of 60° F. by observing the following relations between the atmospheric temperature and the temperature of the water. If the temperatures of the former are respectively 30°, 34°, 38°, 42°, 46°, and 50° F., the temperatures of the circulating water should be 180°, 170°, 160°, 150°, 140°, and 130° F. This is always presuming that the heating service in the wards is sufficient. If this is found not to be the case, the temperature of the water must be raised accordingly. By adherence to a definite scheme of this sort much coal might be saved.—A. B. C.

### Handbook on Radiography.

You will find "Practical Medical Electricity and Radiography," by Alfred C. Norman, M.D., especially useful to house surgeons. It is profusely illustrated, and the price is 5s. net (5s. 5d. post free). The publishers are The Scientific Press, Ltd., 28 and 29 Southampton Street, Strand, London, W.C. 2.—Hospital.

### Tea and Laundry Supply in Sanatorium,

If tea be made in quantities, the usual allowance per head per week is 1½ oz. If individual tea-pots are used you will find it difficult to keep within this allowance, which may be exceeded by an additional ounce per week. The question of washing materials is one that we cannot deal with in the absence of fuller information. It depends upon the type of laundry you have and the number of staff and patients. If you will let us have these details we will endeavour to give you guidance in the matter.—Constant Reader.

